# Critical Conditions Identification for Online Purchase Intention of Fruits: A Fuzzy-Set Qualitative Comparative Analysis

**DOI:** 10.3389/fpsyg.2021.713295

**Published:** 2021-12-02

**Authors:** Can Wang, Fangyu Chen, Feiteng Yi, Yongchang Wei

**Affiliations:** School of Business Administration, Zhongnan University of Economics and Law, Wuhan, China

**Keywords:** online purchase intention, fruit quality, supply service, fruit price concessions, website information quality, perceived risk, fuzzy-set qualitative comparative analysis

## Abstract

With the development of Internet technology, e-commerce platforms have emerged one after another, and the competition of the fruit e-commerce market is increasingly intensified. How to boost customer demand by improving their purchase intention has become a key issue. The study identified the critical conditions leading to high purchase intention of fruits through fuzzy-set qualitative comparative analysis. The empirical analysis was conducted based on an online questionnaire of 344 Internet users. The results reveal that high purchase intention comes from high fruit quality, high fruit price concessions and supply service, and low perceived risk (peripheral conditions). In addition, high purchase intention can also be realized from high fruit quality, high fruit price concessions, high supply service, and high website information quality (peripheral conditions). This study provides more nuanced thinking of how to improve online purchase intention of fruits.

## 1. Introduction

Over the past few years, most consumers buy fruits from online retailers rather than physical stores. The reasons for this change are 2-fold. On the one hand, with the upgrading of mobile Internet technology, the rise of the Internet of things (IoT), cloud computing, and online payment, has brought great convenience to purchasing fruits online and major fruit companies have begun to deploy online retail (Zhao et al., 2021). For instance, Tiantian Orchard which was invested by JD is the first cross-border fresh fruit e-commerce platform in China. Alibaba set up an online fruit retail business in 2017 (Zhang et al., 2021). On the other hand, due to the outbreak of the COVID-19 in December 2019, in response to the country's social distancing policy in China, purchasing vegetables and fruits online has become the first way for residents. This has greatly promoted the development of fruit e-commerce (Chang and Meyerhoefer, 2021). The fruit e-commerce sales in China were about 90 billion by the end of 2019 and had exceeded 100 billion by 2020 (CNNIC, 2021). Thus, fruit e-commerce will develop rapidly in the next few years.

However, despite numerous advantages of online purchase of fruits, attracting consumers to buy fruit is difficult in the highly competitive fruit fresh market. Consumers have many choices and can compare with each other. It becomes a very important issue how to improve the purchase intention of fruits in the fiercely competitive fruit e-commerce market. Many scholars have studied the purchase intention of fruits in different ways. For example, Seo et al. (2020) developed a structural equation model (SEM) to investigate how consumers' purchase intentions are affected by the image and reputation of fresh fruits. Qing et al. (2012) built an SEM to explore the impact of the way of life on Chinese purchase attitude and intention of fresh fruits. Rahmawati et al. (2018) used an SEM to verify the factors influencing the purchase intention of organic fruits. Rodriguez et al. (2017) investigated the effects of attitudes and subjective norms on the purchase intention of fruits of consumers by using the SEM. Meanwhile, Nandi et al. (2017) applied a binomial logistic regression model to explore the intention of consumers for fruits and related affecting factors. Liu et al. (2020) used homogeneity of variance and ANOVA to verify the influence of environmental light on the willingness of consumers to buy fresh agricultural products.

In addition to previous studies on fruit purchase intention, some scholars have also conducted research on the factors that influence fruit online purchase intention. Wei et al. (2018) established an SEM to examine the factors of fruit quality, perceived risk, fruit price concession, and website information quality on fruit online purchase intention. The results revealed that quality and price concessions are the dominant factors affecting the intention of consumers to buy the fruit by taking 344 online consumers as a sample. Xie (2017) developed an SEM to study which risks affect the buying intention of consumers of fresh fruits online. The results indicated that the product risk has the greatest influence on purchase intention. Zhao et al. (2017) used an SEM to explore how reference effects affect consumer's online purchase intention of agricultural products through 297 online consumers from China. The results showed that the reference effect affects online purchase intention through perceived value and perceived risk. Akram et al. (2021) examined the relationship between emotional or rational and online purchase intention by SEM. The results demonstrated that utilitarian and hedonic motivations positively affect online purchase intention. Zhao et al. (2021) applied a logit regression model to assess the impact of consumer attitudes and behaviors on online purchase intention of fresh fruits. Zhang et al. (2021) built SEM to explore the antecedents of online purchase intention based on Technology Acceptance Model.

To sum up, most of the existing studies used regression analysis and SEM to examine the factors that affect fruit purchase intention. They mainly focus on the correlations between different factors or the impact of factors on purchase intention. However, they failed to address the critical conditions, specifically, necessary and sufficient conditions for online purchase intention. Therefore, this study aims at answering the following question: What are the critical conditions for achieving high online purchase intention of fruits? To disclose the answer, we utilized fuzzy-set qualitative comparative analysis (fsQCA) to figure out the configuration of critical conditions in terms of different combinations of factors. It is better than the traditional method that identifies the factors that significantly affect purchase intention. The fsQCA can estimate the effects of different combinations and analyze critical asymmetric relationships (Gligor et al., 2020).

Following this approach, the research contributions of this study are as follows. First, the critical contribution of this study lies in presenting a more nuanced understanding of how to lead to high online purchase intention of fruits. Specifically, fsQCA makes up for the deficiency of multiple linear regression and SEM by examining complex combinatorial connections (Fiss, 2011; Woodside, 2014). It allows for a clearer understanding of the effects of different combinations of antecedents (Woodside, 2015). In addition, the second contribution is to expand the literature with respect to online purchase intention. Compared with previous literature, we explore the factors of purchase intention from a new perspective, which lays a good foundation for future research. Finally, the results are significant to guide management practice. It will cost a lot for managers to satisfy all factors at the same time with limited enterprise resources. Managers, thus, could allocate resources efficiently to achieve effective combinations of antecedents of online purchase intention through research conclusions.

The study is divided into the following sections. The second section reviews the antecedent factors of purchase intention of fruits and the literature on fsQCA. The third section introduces our methods and procedures. Section 4 analyzes the results through empirical analysis. Finally, we summarize the conclusions and discussion of the study.

## 2. Literature Review

### 2.1. Antecedents of Online Purchase Intention of Fruits

**Figure 1:**
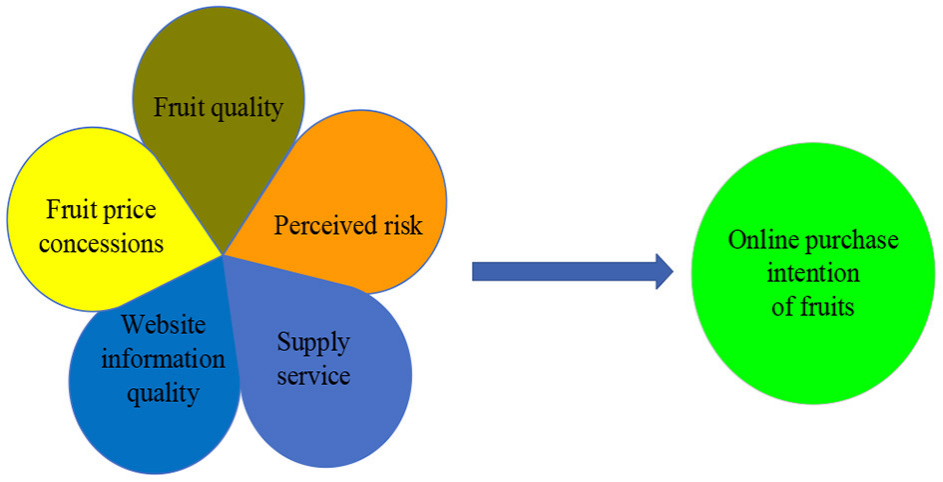
Foundational research model.

Purchase intention is regarded as a kind of cognition or behavior of consumers when they purchase products. In other words, purchase intention to a large extent determines the purchase behavior (Lin and Lu, 2010). Therefore, it is necessary to investigate the factors that affect the willingness of consumers to buy fruits online to stimulate their purchasing behaviors. In the following, we will review the literature related to our study. Based on Wei et al. (2018), a research model (Figure 1) is proposed to examine how five types of factors affect the online purchase intention of fruits.

Online consumers are often uncertain about fruit quality due to the absence of actual viewing and touching. The study has confirmed that the quality of fresh products is considerable for buyers to make purchasing decisions (Hughes and Merton, 1996; Zhang et al., 2020; Zhao et al., 2021). Buyers are willing to spend more money to obtain high-quality fruits. Guo et al. (2012) investigated 350 online shoppers in China and found that product quality was positively correlated to consumer purchase intention. Algharabat and Shatnawi (2014) demonstrated that product quality had a positive impact on purchase intention. Since e-commerce can not bring consumers a real sensory shopping experience, product quality and safety are the predominant factors affecting the participation of consumers in online shopping behavior (Huang et al., 2014; Rosillo-D́ıaz et al., 2019). Fruits are special due to their limited freshness time, so purchasers are always more concerned about the quality and safety of fruits.

Price has always been one of the important elements affecting consumers' purchase intention (Turban et al., 2002; Yadav and Pathak, 2017; Zhao et al., 2021). Many scholars have studied this problem. Kim and Krishnan (2015) selected individual transaction data of the Hmall website and examined the dynamic change of product purchase intention, the results showed that consumers are reluctant to purchase expensive products only through digital information online. Zhang et al. (2018) used the logistic regression method to confirm the purchase intention of consumers was positively affected by a vegetable price-based 840 questionnaires. For a long time, fresh products like fruit have not been widely sold online due to their limited freshness time and inaccessible supply chain technology. At present, the subdivision of the online fruit market has not been fully mature, consumers are still very sensitive to fruit prices (Moon et al., 2008; Wei et al., 2018). Hence, attractive price is particularly important in competing with traditional markets and seizing market share for fruit e-commerce.

Supply service refers to the whole process from placing an order, receiving products, and returning products. As online shopping is becoming more and more common, the supply service is becoming a key factor influencing online purchase intention (Zhao et al., 2021). High quality of supply service can eliminate the shopping scruples of consumers as much as possible and stimulate purchase intention (Xu et al., 2017). Cahyaningrum et al. (2020) found that supply chain integrity can strengthen the trust of customers in online purchasing. Bienstock and Royne (2010) found that supply service quality is significantly correlated with online purchase intention. Since fruits have a short shelf life and will deteriorates over time, convenient supply services and sound packaging have a direct impact on the purchase experience of this consumer.

A well-designed website with valid information can greatly facilitate the completion of transactions. Turban et al. (2002) deemed that the accuracy and completeness of website information are very significant for consumers to choose consumption platforms in such a competitive environment, and this viewpoint has been proved in many actual situations, such as apparel retailing and air ticket (Kim and Niehm, 2009; Mohd Sam and Tahir, 2009). Gao et al. (2012) explored the interaction effect of quantity and quality of web information with unconscious thought mode. The results showed that the website information quality is more likely to determine consumer purchasing intention. Indeed, people own limited information processing abilities. We often feel confused when facing mass information beyond our processing capacity, a well-designed website with approximate valid information can greatly enhance the online shopping experience and degree of satisfaction of consumers (Zhang et al., 2021; Zhao et al., 2021). Furthermore, some researchers have devoted themselves to the influence of website interface characteristics on online purchase intention and proposed specific recommendations for website design to improve online purchasing intention (Song and Zahedi, 2005; Hausman and Siekpe, 2009; Dedeke, 2016).

Online shopping is realized by placing orders online and delivering goods offline, you cannot see or touch the real products until you get them. Compared with traditional fruit buying channels, consumers would pay more attention to potential safety hazards when purchasing fruit online (Rosillo-D́ıaz et al., 2019). Fruit is a product that cannot be tasted before buying, which makes consumers very sensitive to the risks of buying fruit online. Ventre and Kolbe (2020) used SmartPLS to explore the influence of perceived risk on online purchase intention. Liao et al. (2021) found that perceived risk negatively affects online purchase intention. Due to the fact that we are stepping into the digital age, there exists a lot of uncertainty, consumers may concern about the privacy disclosure and online payment security, which will greatly reduce online purchase intention of fruits (Ariffin et al., 2018; Wei et al., 2018; Sharma et al., 2021).

### 2.2. The Fuzzy-Set Qualitative Comparative Analysis

The fsQCA endeavor to distinguish common designs of affiliations between sets of factors and explanations that can be applied to all contexts (Amara et al., 2020). It is very suitable to use fsQCA to seek the antecedent combinations of the willingness to buy fruit online. First and foremost, the fsQCA identifies all combinations of conditions that lead to the same result (Eng and Woodside, 2012; Woodside et al., 2012). This accepts that particular case factors impact ahead a particular result relies on how it interacts with others, instead of on the quality for its single person association for different variables. In addition, unlike other related methods, the fsQCA has no strict requirement on sample size (Ragin, 2006; Fiss, 2011).

The fsQCA was developed by Ragin (2000). Since then, the fsQCA has been widely used in recent years. For instance, Urueña and Hidalgo (2016) used fsQCA to test antecedent factors of loyalty in complaint behavior, while Chaparro-Peláez et al. (2016) used the method to analyze the impact of motivations and barriers on online-shopping behavior. Fiss (2011) utilized the fsQCA to analyze the high-technology enterprises. Amara et al. (2020) used fsQCA to evaluate the research efficiency of scholars from Canadian. Finally, Gligor et al. (2020) applied fsQCA to explore supply chain strategy.

## 3. Methods and Procedures

### 3.1. Data Collection

An online questionnaire was designed for this empirical analysis which refers to Wei et al. (2018). The questionnaire includes respondent basic information and the scale measurement. The basic information of the respondents is shown in Table 1. About 125 men (36.3%) and 219 women (63.7%) were surveyed. In terms of education experience, more than 80 percent of respondents have a master's degree or bachelor's degree. All of the respondents had an online shopping experience, but 189 respondents do not have an online purchase experience of fruits. Meanwhile, all construct variables were measured using a 7-point Likert scale (1-strongly disagree 7-strongly agree). All of the respondents were asked to indicate their degree of agreement with each statement. The study distributed 360 questionnaires through WeChat, E-mail, and professional websites. In all, 359 questionnaires were successfully retrieved, 15 invalid questionnaires were removed due to incomplete data, and 344 valid questionnaires were obtained (constituting a 95.6% response rate).

**Table 1:** Sample characteristics.

**Attributes**	**Items**	**Frequency**	**Percent (%)**
Gender	Male	125	36.3
	Female	219	63.7
	16–20	47	13.7
	21–25	224	65.1
Age	26–30	43	12.5
	30–35	18	5.2
	Over 35	12	3.5
	Junior college	21	6.1
	College	37	10.8
Education	Undergraduate	183	53.2
	Master	97	28.2
	Ph.D	6	1.7
	Student	209	60.8
	Enterprise employee	70	20.3
Career	Government staff	11	3.2
	Staff in medical or educational institutions	14	4.1
	Other occupations	40	11.6
Purchase fruit experience	Yes	155	45.1
	No	189	54.9

In addition, after years of rapid development, e-commerce in China is now very mature. According to the data from CNNIC (China Internet Network Information Center), the online shopping users of China had reached 710 million, accounting for 78.6% of the total number of Internet users by 2020, and this proportion is higher among young people. Most of the respondents to our questionnaire belong to the younger generation, almost all of whom have an online shopping experience. Therefore, we believe that the respondent of the questionnaire is reasonable.

### 3.2. Common Method Bias

Harman's one-factor test was used to evaluate common method bias (Podsakoff and Organ, 1986; Hochwarter et al., 2004). The exploratory factor analysis was used to extract a principal component and the variance explanation rate was 45.35% (i.e., lower than 50%) of the total variance and the model fitting was poor [root-mean-square error of approximation (RMSEA) = 0.153, comparative fit index (CFI) = 0.723, Tucker-Lewis index (TLI) = 0.683, goodness-of-fit index (GFI) = 0.690, adjusted goodness-of-fit index (AGFI) = 0.601] (Kline, 2015). When we did not limit all items to one factor, the model fitting was better (RMSEA = 0.067, CFI = 0.953, TLI = 0.939, GFI = 0.916, AGFI = 0.876). The first factor explained 24.02% of the variance (i.e., lower than 50%). This evidence indicates that common method bias will not pose a threat to the study.

### 3.3. Validity and Reliability Test

Before fsQCA analysis, it is critical to test the validity and reliability of all variables. SPSS 22.0 and AMOS 21.0 were used to analyze the reliability and validity of the scale. The factor loadings of all the explicit variables should be above 0.7 (Hair et al., 1998), and loadings greater than 0.6 are within the acceptable range (Vinzi et al., 2010). Most of the factor loadings in this study are greater than 0.70, except for one of the supply services and one of the perceived risk indicators, with values of is 0.664 and 0.655, respectively. From these results, we can deem that all variables are valid.

Discriminant validity was analyzed by average variance extracted (AVE). The values of AVE should be above 0.5 (Hair et al., 1998). We can see that the AVE values of all variables are greater than 0.50 from Table 2. Meanwhile, as shown in Table 3, the minimum square root of the AVE is 0.743 and over the correlations between constructs (Fornell and Larcker, 1981), which confirm that the validity of each construct is brilliant (Kline, 2015). Table 2 shows that the values of Cronbach's alpha of all variables are above 0.70, and values of composite reliability are above 0.70 (Fornell and Larcker, 1981; Hair et al., 1998).

**Table 2:** Measurement items and reliability results.

**Construct**	**Items**	**STD**	**Cronbach's α**	**CR**	**AVE**
	Q9	0.781	0.862	0.865	0.682
Purchase intention	Q10	0.889			
	Q11	0.804			
	Q12	0.869	0.879	0.881	0.713
Fruit quality	Q13	0.893			
	Q14	0.767			
	Q15	0.710	0.814	0.815	0.596
Fruit price concessions	Q16	0.754			
	Q17	0.846			
	Q18	0.664	0.773	0.786	0.552
Supply service	Q19	0.780			
	Q20	0.780			
	Q21	0.759	0.856	0.861	0.675
Website information quality	Q22	0.875			
	Q23	0.826			
Perceived risk	Q26	0.655	0.758	0.781	0.648
	Q27	0.931			

**Table 3:** Means, SD, and interrelations of variables.

**Variable**	**Mean**	**S.D**.	**(1)**	**(2)**	**(3)**	**(4)**	**(5)**	**(6)**
Purchase intention(1)	4.164	1.167	**0.826**					
Fruit quality(2)	3.936	1.147	0.689^**^	**0.844**				
Fruit price concessions(3)	4.319	1.153	0.551^**^	0.532^**^	**0.772**			
Supply service(4)	4.347	1.085	0.551^**^	0.688^**^	0.576^**^	**0.743**		
Website information quality(5)	4.417	1.079	0.476^**^	0.610^**^	0.488^**^	0.762^**^	**0.822**	
Perceived risk(6)	5.015	1.111	–0.067	–0.140^**^	0.078	0.001	0.028	**0.805**

** *p < 0.01. The diagonal lines and bold values are represent the square root of average variance extracted (AVE)*.

In addition, AMOS 21 was used for confirmatory factor analysis. From the results, the fitting indexes of the measurement model are λ^2^/*df* = 2.550, RMSEA = 0.067, GFI = 0.916, normed fit index (NFI) = 0.926, CFI = 0.953, incremental fit index (IFI) = 0.954, RFI = 0.903, parsimonious normed fit index (PNFI) = 0.708, parsimonious goodness-fit-index (PGFI) = 0.623, and adjusted goodness-of-fit index (AGFI) = 0.876. All the indicators are within the critical criteria (MacCallum et al., 2006; Wang et al., 2016). This shows that the fitting relationship between the measurement model and the data is better.

### 3.4. Descriptive Analysis

The mean, SD, and correlation coefficient of each variable analyzed by SPSS 22 are shown in Table 3. The results reveal that the four variables, such as fruit quality (β = 0.689, *p* < 0.001), fruit price concessions (β = 0.551, *p* < 0.001), supply service(β = 0.551, *p* < 0.001), and website information quality (β = 0.476, *p* < 0.001) have significant influence on purchase intention. The influence coefficient of perceived risk on purchase intention is –0.067, and the *p*-value is 0.218, which indicates that the influence is not significant. However, this study also includes it to carry out fsQCA for some reasons. First, some scholars have confirmed that perceived risk has a certain influence on buying intention (Cheung et al., 2005; Ariffin et al., 2018). Second, there exist some risks due to the immaturity of Internet technology. Meanwhile, there exists a lot of uncertainty, because the fruits can not be touched or tasted until it is delivered to the consumer.

### 3.5. The fsQCA Procedure

The fsQCA includes two steps: data calibration and truth table construction. Converting the data into fuzzy sets is important because results strongly depend on the calibration. All the values of fuzzy-sets range from 0.00 to 1.00 in each condition. This study used the fsQCA 3.0 software program for an automatic calibration procedure (Ragin, 2007). During the data calibration, three breakpoints need to be set, 0.05 refers to the full non-membership threshold, 0.50 refers to the crossover point, and 0.95 refers to the full membership threshold. In this study, we used 7 to represent full membership in a category, 1 to represent full non-membership in a category, and 4 to represent the maximum uncertainty of a certain category of membership (Leischnig et al., 2014). Meanwhile, for the calibration of the perceived risk, we utilized 5 to represent maximum uncertainty membership in a category because the mean value of risk variables is 5.01, which is higher than other variables. Table 4 shows the calibration anchor points.

**Table 4:** Calibration anchor points.

**Variable**	**Anchor points**		
	**full membership**	**maximum uncertainty**	**non-membership**
Purchase intention	7	4	1
Fruit quality	7	4	1
Fruit price concessions	7	4	1
Supply service	7	4	1
Website information quality	7	4	1
Perceived risk	7	5	1

After each variable was calibrated to the set members, fsQCA 3.0 used the truth table algorithm to obtain different combinations of conditions that produce high online purchase intention. The fsQCA 3.0 built a truth table that lists all possible combinations, of causal conditions, each with a value of 1 or 0 (Fiss, 2011). The value of 1 indicates a high purchase intention, while the value of 0 indicates a low purchase intention. This study includes five causal conditions. The truth table will contain 32 latent configurations in which each sample will be assigned to 32 rows. In addition, the fsQCA minimizes the number of causal conditions through Boolean algebra. If two conditions A and B, and condition A and not B can cause C, then Boolean algebra shows that A can cause C (Ragin, 2007). The intermediate solution, parsimonious solution and complex solution, are obtained through many counterfactual analyses. Finally, the core conditions and peripheral conditions can be found by analyzing the intermediate solution and the parsimonious solution.

## 4. Results

### 4.1. Analysis of Necessary Conditions

The fuzzy-set qualitative comparative analysis includes the inspection of necessary and sufficient conditions. If a condition always occurs when a certain result exists, then this condition is the necessary condition for the existence of the result. In a nutshell, without this condition, the result cannot be produced. The necessary conditions for high purchase intention and low purchase intention are shown in Table 5. An important indicator to measure necessary conditions is consistency. Following the suggestions from Schneider and Wagemann (2012), the consistency score of necessary conditions should be above 0.9. In terms of high purchase intention, there is no condition that meets the criteria, which shows the absence of necessity conditions of high purchase intention. However, the consistency score of low fruit quality is 0.905. Thus, low fruit quality is a necessary condition for low purchase intention.

**Table 5:** Analysis of necessary conditions.

**Condition**	**High purchase intention**		**Low purchase intention**	
	**Consistency**	**Coverage**	**Consistency**	**Coverage**
Fruit quality	0.830	0.909	0.684	0.659
~Fruit quality	0.688	0.712	0.905	0.824
Fruit price concessions	0.877	0.822	0.760	0.848
~Fruit price concessions	0.603	0.740	0.785	0.848
Supply service	0.885	0.823	0.775	0.634
~Supply service	0.606	0.754	0.783	0.856
Website information quality	0.887	0.810	0.798	0.641
~Website information quality	0.607	0.773	0.763	0.856
Perceived risk	0.765	0.733	0.829	0.698
~Perceived risk	0.685	0.820	0.683	0.719

*~ denotes the low levels of a condition*.

### 4.2. Analysis of Sufficient Conditions

The fsQCA method will generate a complex solution, intermediate solution, and parsimonious solution. The complex solution is the most rigorous, but the conclusions are often more complicated. The parsimonious solution is the most relaxed, but the conclusions are often too simple, and many conclusions may conflict with the actual situation, so its enlightenment is poor. The intermediate solution combines the theoretical knowledge of the researcher and the analysis of the case, and the conclusions are enlightening and universal. Therefore, most scholars choose the intermediate solution in their research (Ragin, 2014). This study selects intermediate solutions for reporting. In addition, this study uses the classification of conditions of Fiss (2011), defining all conditions that appear in the parsimonious solution as core conditions and all conditions that appear in the intermediate scheme but are excluded by the parsimonious solution as peripheral conditions.

The sufficiency analysis of the conditional configuration is the core of the fsQCA method, which mainly analyzes the sufficiency of the configuration formed by different antecedent conditions to the result. In the next step, we will construct a truth table to analyze the sufficient condition relationship (Ragin, 2008). Same as previous studies, the consistency threshold was set at 0.8, which is over the least suggested score of 0.75 (Fiss, 2011; Woodside and Baxter, 2013). We also set the minimum coverage of each configuration to be greater than 0.10 (Woodside et al., 2018).

The results of the sufficient conditions of high purchase intention and low purchase intention are shown in Table 6. Each column represents a combination of conditions that produce a particular result. In order to present the results of fuzzy-set analysis, the study uses black circles (•) to describe high-level conditions and cross circles (⊗) to describe low-level conditions. Larger circles represent core conditions and smaller circles represent peripheral conditions (Misangyi and Acharya, 2014; Gligor et al., 2020).

**Table 6:** Analysis of sufficient conditions.

**Conditions**	**Outcome**		
	**High purchase intention**		**Low purchase intention**
	**Solution 1**	**Solution 2**	**Solution 3**
Fruit quality	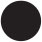	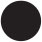	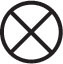
Fruit price concessions	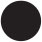	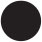	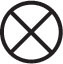
Supply service	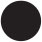	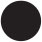	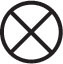
Website information quality		•	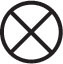
Perceived risk	⊗		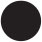
Consistency	0.964	0.943	0.956
Raw coverage	0.586	0.741	0.579
Unique coverage	0.008	0.162	0.579
Overall solution consistency	0.941		0.956
Overall solution coverage	0.748		0.579

*The black circles (•) represents high-level conditions and cross circles (⊗) represents low-level conditions. Larger circles represent core conditions and smaller circles represent peripheral conditions*.

As shown from Table 6, Solution 1 indicates that high purchase intention can come from high fruit quality, high fruit price concessions and supply service, and low perceived risk (peripheral conditions). The consistency of this solution is 0.964, which explains many situations with a raw coverage rate of 0.586. Solution 2 shows that high fruit quality, high supply service and fruit price concessions, and high website information quality (peripheral conditions) lead to high purchase intention. Among the three solutions, Solution 2 has the highest raw coverage rate, also reaching 0.7 (consistency = 0.943). Solution 3 appears that the combination of low fruit quality, low fruit price concessions, low supply service and website information quality, and high perceived risk result in low purchase intention (consistency = 0.956 and raw coverage = 0.579).

### 4.3. Robustness Test

Two aspects of robustness tests are carried out in the study. Firstly, the consistency threshold was increased to 0.85 and 0.9, the calculations were performed separately, and the calculation results are consistent with those in Table 6. Second, when the frequency threshold was changed to 2 and 3, respectively, a sufficient condition was obtained for high purchase intention, which corresponds to Solution 2 in Table 6. However, the results are the same as Table 6 for low purchase intention. Hence, the results of this study are stable and dependable.

## 5. Discussion and Conclusions

This study used fsQCA to examine the conditions leading to high purchase intention and low purchase intention, respectively. The critical advantage of using fsQCA is that it is the possibility to find new configurations leading to high purchase intention and low purchase intention. The results will not only help managers to efficiently allocate resources, but also promote the sustainable development of fruit e-commerce.

### 5.1. Theoretical Contributions

First, this study enriches online purchase intention literature and verifies that e-commerce platforms do not need to improve all conditions to achieve high purchase intention. Wei et al. (2018) confirmed that fruit quality and fruit price concessions have a marked impact on the willingness of consumers to buy online. Zhao et al. (2017) found perceived risk mediating the relations between reference effects and online purchase intention. We believe that there will be a high level of purchase intention when all conditions are at a high level. However, due to the limited resources of e-commerce platforms, it is impossible to take into account all factors at the same time. Instead, resources can only be reasonably allocated according to the importance of factors.

Second, the three solutions provide a more detailed understanding of the online purchase intention of fruits. These solutions indicate that perceived risk and website information quality are not core conditions. In other words, e-commerce platforms do not always need to possess a high level of website information quality and a low level of perceived risk. In fact, the perceived risk of customers to some extent comes from e-commerce sites. With the continuous development of Internet technology, website security performance is gradually improved, as long as it can meet the normal shopping of consumers, it will not bring them a lot of trouble.

Third, the study complements shortcomings of regression and SEM (Gligor et al., 2020). The fsQCA develops the types of research problems that scholars can solve and captures the overall characteristics and results of the system through the abstraction of the system without describing all its parts, thereby obtaining more subtle discoveries. Importantly, we believe that fsQCA can complement these methods and provide additional insights into the phenomenon of online purchase intentions.

### 5.2. Managerial Implications

In this research, we found that low fruit quality is a necessary condition for low purchase intention. It indicates that fruit quality is the most critical factor when people purchase fruit online. However, the quality of the fruit alone is not enough. There are two combinations that can lead to high purchase intention. Solution 1 in Table 6 shows that high purchase intention comes from high fruit quality, high fruit price concessions and supply service, and low perceived risk (peripheral conditions). Solution 2 indicates that high fruit quality, high fruit price concessions and supply service, high website information quality (peripheral conditions) lead to higher purchase intention. It suggests that high fruit quality, fruit price concessions and high supply service are both the necessary conditions and the core conditions for high purchase intention, which is consistent with the results in the literature (Shih, 2004; Wei et al., 2018). Price and quality have always been important factors considered by consumers. Due to the rapid development of the supply chain, supply services have gradually become one of the important factors considered by consumers. The study in Wei et al. (2018) found that website information quality and perceived risk have no significant effect on online purchase intention of fruits. This is slightly different from our conclusions. Our results show that the high website information quality and low perceived risk are the peripheral conditions of high purchase intention but not the core conditions. In addition, this study also analyzed the condition of low purchase intention. The solution shows that the combination of low fruit quality and fruit price concessions, low supply service and quality of website information, and high perceived risk result in low purchase intention. High perceived risk is a core condition of low purchase intention which is the same as the study in Cheung et al. (2005). This suggests that consumers may also be affected by risks, for instance, privacy disclosure, property security, and advertising harassment when purchasing fruit online.

According to the results of the research, the following helpful suggestions are provided to fruit e-commerce enterprises. First and foremost, the high quality of fruit is the most important factor. It directly determines whether consumers are willing to buy. Fruit e-commerce should ensure the freshness and health of fruit with limited resources. In addition, we should also attach importance to supply services and price concessions. On the one hand, we can carry out more preferential activities to provide consumers with cheaper prices. On the other hand, we should improve the supply service and deliver fruits to consumers faster. Last but not least, website information quality and risks cannot be ignored. E-commerce websites provide consumers with all kinds of information about fruits, which is convenient for consumers to browse and place orders. Therefore, it is very important to maintain the website information and provide convenience for consumers to buy fruits. Meanwhile, in order to prevent online payment risk, the website should strengthen security and ensure the personal information and transaction information of consumers.

### 5.3. Limitations and Future Research

There are some limitations that highlight the need for future research. First, we only consider some conditions from existing literature while ignoring other conditions, such as perceived value and emotions. Second, the sample source of this study is single. Expanding the sample in different ways would certainly make results more generalizable. In the future, we could further analyze more conditions through samples from different sources.

## Data Availability Statement

The original contributions presented in the study are included in the article/Supplementary Material, further inquiries can be directed to the corresponding author.

## Ethics Statement

Ethical review and approval were not required for the study on human participants in accordance with the local legislation and institutional requirements. Written informed consent from the patients/participants was not required to participate in this study in accordance with the national legislation and the institutional requirements.

## Author Contributions

YW and FC developed the conceptual framework and revised the whole manuscript. CW analyzed the data and wrote the manuscript. FY collected the data and discussed the results. All authors contributed to the article and approved the submitted version.

## Funding

This study was supported by the MOE (Ministry of Education in China) Project of Humanities and Social Sciences (No. 21YJA630094), the Teaching and Research Project of Zhongnan University of Economics and Law in 2018 (No. YB2018019), and the Fundamental Research Funds for the Central Universities, Zhongnan University of Economics and Law (No. 202111011).

## Conflict of Interest

The authors declare that the research was conducted in the absence of any commercial or financial relationships that could be construed as a potential conflict ofinterest.

## Publisher's Note

All claims expressed in this article are solely those of the authors and do not necessarily represent those of their affiliated organizations, or those of the publisher, the editors and the reviewers. Any product that may be evaluated in this article, or claim that may be made by its manufacturer, is not guaranteed or endorsed by the publisher.
